# Opioid-free anesthesia in oncologic surgery: the rules of the game

**DOI:** 10.1186/s44158-022-00037-8

**Published:** 2022-02-05

**Authors:** Alessandro De Cassai, Federico Geraldini, Serkan Tulgar, Ali Ahiskalioglu, Edward R. Mariano, Burhan Dost, Pierfrancesco Fusco, Gian Marco Petroni, Fabio Costa, Paolo Navalesi

**Affiliations:** 1 UOC Anaesthesia and Intensive Care Unit, University Hospital of Padua, Via Giustiniani 1, 35127 Padua, Italy; 2 Samsun University Faculty of Medicine, Training and Research Hospital, Samsun, Samsun Turkey; 3 Department of Anesthesiology and Reanimation, Ataturk University Faculty of Medicine, Erzurum, Turkey; 4 Clinical Research, Development and Design Application and Research Center, Ataturk University School of Medicine, Erzurum, Turkey; 5 Anesthesiology and Perioperative Care Service, Veterans Affairs Palo Alto Health Care System, Palo Alto, CA USA; 6 Department of Anesthesiology, Perioperative and Pain Medicine, Stanford University School of Medicine, Stanford, CA USA; 7 Department of Anesthesiology and Reanimation, Ondokuz Mayis University Faculty of Medicine, Samsun, Turkey; 8 Department of Anesthesia and Intensive Care Unit, San Salvatore Academic Hospital of L’Aquila, L’Aquila, Italy; 9 Department of Life, Health and Environmental Sciences, University of L’Aquila, L’Aquila, Italy; 10 Unit of Anaesthesia, Intensive Care and Pain Management, Department of Medicine, Università Campus Bio-Medico di Roma, Rome, Italy; 11 University of Padova, Department of Medicine, Padua, Italy

**Keywords:** Opioids, Anesthesia, Cancer, Review

## Abstract

**Background:**

Opioids are frequently used in the postoperative period due to their analgesic properties. While these drugs reduce nociceptive somatic, visceral, and neuropathic pain, they may also lead to undesirable effects such as respiratory depression, urinary retention, nausea and vomiting, constipation, itching, opioid-induced hyperalgesia, tolerance, addiction, and immune system disorders. Anesthesiologists are in the critical position of finding balance between using opioids when they are necessary and implementing opioid-sparing strategies to avoid the known harmful effects. This article aims to give an overview of opioid-free anesthesia.

**Main body:**

This paper presents an overview of opioid-free anesthesia and opioid-sparing anesthetic techniques. Pharmacological and non-pharmacological strategies are discussed, highlighting the possible advantages and drawbacks of each approach.

**Conclusions:**

Choosing the best anesthetic protocol for a patient undergoing cancer surgery is not an easy task and the available literature provides no definitive answers. In our opinion, opioid-sparing strategies should always be implemented in routine practice and opioid-free anesthesia should be considered whenever possible. Non-pharmacological strategies such as patient education, while generally underrepresented in scientific literature, may warrant consideration in clinical practice.

## Introduction

Moderate to severe pain is reported to occur in 30–80% of patients on the first postoperative day [1]. If postoperative pain is not appropriately managed, it may lead to central sensitization and chronic postsurgical pain [2]. Opioids are frequently used in the postoperative period due to their analgesic properties. While these drugs reduce nociceptive somatic, visceral, and neuropathic pain, they may also lead to undesirable effects such as respiratory depression, urinary retention, nausea and vomiting, constipation, itching, opioid-induced hyperalgesia, tolerance, addiction, and immune system disorders [3]. Moreover, excessive prescribing has contributed to the opioid epidemic that has primarily affected people in North America [4, 5]. Enhanced Recovery After Surgery (ERAS) protocols recommend avoiding long acting or high doses of opioids in order to reduce their side effects in the postoperative period [6, 7].

Anesthesiologists are therefore in the critical position of balancing the judicious use of opioids when they are indicated while avoiding opioid-related harm and effectively managing pain. There are a number of methods or approaches that can be used to reduce opioid use before, during, and after surgery. These include identifying and optimizing individuals at risk of long-term opioid dependence and using a multimodal non-opioid analgesic regimen perioperatively [8, 9]. In this framework, opioid-free anesthesia (OFA) may be an interesting approach to cancer patients and is an active field of research. This article aims to give an overview of OFA and of opioid-sparing strategies more generally. Both the pharmacological and non-pharmacological strategies will be discussed, highlighting possible advantages and drawbacks of these approaches.

## Main text

### Rationale

Cancer is one of the most common causes of mortality worldwide leading to an estimated 10 million deaths in 2020 [10]. Surgery is an important element of treatment in these patients. With the emergence of new surgical and technological developments, the frequency of oncological surgeries is also increasing. Analgesia management in oncological surgery differs in some aspects when compared to other surgical procedures, as this group of patients are frequently prescribed opioids during the preoperative period for the management of cancer-related pain. Also, malnutrition, depression secondary to chronic illness and other physiological changes affect the pharmacokinetics of drugs in general. Opioids have been associated with several problems: dose-dependent side effects that may be debilitating and delay rehabilitation; negative impact on nociceptive pathways with dose-dependent hyperalgesia and insufficient efficacy in reducing incident pain. Moreover, many patients among the broad surgical population may have a considerable advantage from OFA, in particular morbidly obese, patients with sleep apnoea and/or chronic obstructive pulmonary disease, and the acute/chronic opioid addicted [11]. Opioids are also thought to interfere with the immune system which may have a negative impact on infectious or cancerous diseases; opioids might bind to opioid receptors expressed by tumor cells; thus, opioid use in oncological patients may favor tumor angiogenesis, growth, and spreading of micrometastasis. Hence, cancer patients may take the greatest advantage from this technique [12].

OFA technique is feasible following the basic concept of multimodal analgesia that one drug alone cannot completely replace opioids. An adequate association of drugs, possibly with the concurrent use of regional anesthesia techniques, permits a good quality of anesthesia without using opioids. The multimodal approach blocks or mitigates the transmission of pain and inflammatory pathways at different levels, reducing or eliminating (in the case of OFA) opioid requirements [13].

OFA has been shown to be feasible but only few well-designed studies have been published so far and very little data is available on long-term outcomes (incidence of hyperalgesia and persistent pain; opioid abuse; cancer recurrence and survival outcomes). The overall literature remains scarce and whether OFA is clinically advantageous or not in the different fields of application and with different modalities is yet to be established.

The use of OFA techniques may be appropriate in certain surgeries for cancer patients, especially if regional anesthesia and more generally opioid-sparing strategies can be employed. Preclinical trials have suggested that opioids inhibit the function of natural killer cells and increase cancer recurrence by affecting angiogenesis and tumor cell signaling pathways. On the other hand, regional anesthesia is thought to contribute positively to outcomes in cancer patients by reducing surgical stress response and thus reducing the need for volatile anesthetics and opioids [14]. However, a meta-analysis showed insufficient evidence to recommend any analgesia technique for patients undergoing cancer surgery [15]. The popularity of OFA has increased among anesthesiologists worldwide recently.

However, differences in definitions have led to conflicting results. Its benefits, limitations, and applicability have been questioned, with some studies reporting that it does not provide any benefits [9]. However, in a meta-analysis, strict opioid-free anesthesia was found to significantly reduce postoperative morphine consumption and postoperative adverse events in selected surgeries without increasing intraoperative adverse events, postoperative pain, and discharge time from the post-anesthesia care unit [16]. Although there are many studies regarding OFA techniques related to drugs and regional and non-pharmacological options, meta-analyses have failed to report solid recommendations or suggestions. It is obvious that further studies are required to determine whether patient characteristics should be used to create individualized analgesia regimens or if a common single modality should be adopted for a majority of or for all patients.

### Intravenous agents

Dexmedetomidine is a potent and highly selective α-2 adrenoreceptor agonist with sedative, anxiolytic, sympatholytic, and opioid-sparing properties. It reduces the need for anesthetics and opioids without causing respiratory depression but existing literature has reported conflicting results [17]. Many studies have already shown that OFA allows opioid sparing in the postoperative period. After bariatric [18] and spine surgery [19], OFA techniques led to lower morphine consumption and better postoperative analgesia. Liu et al. showed that dexmedetomidine may be a favorable anesthetic adjuvant in breast cancer surgery [20]. Dexmedetomidine has a range of perioperative applications, from reducing the hemodynamic response to tracheal intubation [21] to reducing the incidence of postoperative nausea and vomiting (PONV) [22]. In a meta-analysis, Grape et al. reported that dexmedetomidine was superior when compared to intraoperative remifentanil, with better pain outcomes postoperatively in the first 24 h. In addition, dexmedetomidine has been linked to fewer episodes of hypotension, shivering, and PONV [17]. On the other hand, Beloeil H et al. reported higher rates of severe bradycardia with the use of dexmedetomidine-based, opioid-free anesthesia [23]. In addition, clinically significant hypotension, delay in discharge, and prolongation of hypoxia may be observed with dexmedetomidine infusion [9].

Ketamine, an N-methyl-d-aspartate (NMDA) receptor antagonist, is widely known as a dissociative anesthetic and phencyclidine derivative. It has complex hypnotic, analgesic, antidepressant and psychotomimetic effects that cannot be explained only by NMDA and receptor antagonism. Ketamine has also been shown to bind to all types of opioid receptors with the highest affinity for mu, kappa, and delta receptors [24]. Ketamine has a unique profile combining analgesia and sedation by dissociative effect. Its analgesic effect is dose dependent, and intravenous ketamine is an effective adjuvant for postoperative analgesia [25]. Ketamine leads to effective analgesia at subanesthetic doses (≤ 0.3 mg/kg intravenous) [26]. However, it should be cautiously used as only a single dose can lead to hallucinations and nightmares. Although the mechanism of opioid-induced hyperalgesia is not fully known, high activation and stimulation of NMDA receptors are among the leading hypotheses. Opioid-induced hyperalgesia is more common in patients using high-dose opioids for the treatment of cancer pain and ketamine is often used in the treatment of this condition given its effect on NMDA receptors [27]. Ketamine is in racemic form containing equal proportions of R-ketamine and S-ketamine. S-ketamine has higher affinity for NMDA receptors than R-ketamine, its anesthetic potency is 3-4 times higher and undesirable side effects are reduced [28]. There is evidence that ketamine infusion is useful for the management of chronic pain [29].. Because of these beneficial properties, the use of ketamine in cancer patients is increasing. Lidocaine is an amide group local anesthetic that is used clinically for analgesia. Infusion in the perioperative period is safe and has clear advantages, such as reduced postoperative pain, decreased length of hospital stay and PONV and perioperative opioid requirements as well as accelerated postoperative recovery of bowel function [25–32]. Perioperative intravenous lidocaine has been shown to have a positive effect on cancer outcomes in clinical studies. Additionally, the mechanism of this effect has been demonstrated in in vitro and animal studies [33]. In patients undergoing pancreatectomy, Zhang et al. found that those who received intravenous lidocaine preoperatively had a considerably improved overall survival [34]. Additional intravenous agents such as magnesium, steroids, β-blockers, and oral pregabalin have been shown to have beneficial effects in OFA, though more studies are needed for their use to become routinely part of OFA [9, 35–37].

### Regional anesthesia

The use of regional anesthesia applications provides many essential benefits such as lower pain scores in the postoperative period, early hospital discharge, reduction in the incidence of PONV, and reduction in the use of additional analgesics. In addition, regional anesthesia is not only an important component of pain management: evidence suggests that it may also play a role in inhibiting cancer progression [38, 39].

The importance of regional anesthesia in accepting an opioid-free modality was demonstrated in a prospective study of 2382 upper and lower extremity peripheral nerve blocks, with 90% of patients not requiring opioids in the PACU postoperatively [40]. Therefore, regional anesthesia applications can be considered the cornerstone of OFA. Another entity is that inadequately treated acute postoperative pain may be associated with 10–50% of persistent postsurgical pain [41]. This is a significant contributor to long-term opioid overuse. A meta-analysis examined the effect of regional anesthetic techniques on the incidence of persistent postoperative pain. This meta-analysis found that regional anesthesia techniques significantly reduced the risk of persistent postoperative pain development compared to standard analgesia in breast surgery, thoracotomy, and cesarean section [42].

Epidural analgesia, thoracic paravertebral block, intercostal block, local infiltration analgesia, and transversus abdominis plane block are the most known and effective opioid-free regional anesthesia techniques [43]. Neuraxial analgesia minimizes perioperative opioid consumption in urological, thoracic, obstetric, orthopedic, and major abdominal surgeries. Epidural analgesia and continuous catheter techniques are used quite frequently, especially in open abdominal and thoracic surgeries [44]. Recently, a new horizon has been opened in OFA with the introduction of ultrasonography in regional anesthesia practice. It is claimed that not only neuraxial techniques but also interfacial plane blocks contribute positively to the opioid-free or opioid-sparing effect. Interfacial plane blocks may be a feasible option, especially in cases where neuraxial techniques cannot be applied or are contraindicated. Although there is not enough evidence yet, it has been shown in randomized controlled studies and case reports that interfacial plan blocks reduce opioid consumption or have an opioid-free anesthesia effect [45–47]. In a study comparing TAP block with liposomal bupivacaine and epidural analgesia, TAP block was shown to be an alternative to epidural analgesia for pain control in patients undergoing abdominal surgery [48]. TAP and quadratus lumborum blocks can be good alternatives in abdominal and obstetric surgeries, while pectoral and serratus plane blocks find use in breast and thoracic surgeries [49]. In thoracic and lumbar surgeries, erector spinae plane and thoracolumbar interfascial plane blocks are also potentially interesting techniques with opioid-sparing effects [50, 51]. Another advantage of interfacial plane blocks in the opioid-free concept is the opportunity of inserting an indwelling catheter to maintain a continuous analgesic infusion.

### Non-pharmacological treatment

In the setting of opioid-free analgesia, optimizing perioperative care with non-pharmacological strategies to mitigate nociception is a potentially understudied strategy, with literature currently dominated by studies on pharmacological interventions. Fiore et al., in a scoping review conducted in 2019, identified only 13 studies addressing specifically non-pharmacological interventions in opioid-free analgesia [52].

Transcutaneous electrical nerve stimulation *(*TENS) is one of the most studied non-pharmacologic strategies to improve analgesia in surgical patients and is used for pain management of both acute and chronic pain irrespective of cause. Though its role in postoperative analgesia may be yet controversial [53], TENS was found to substantially reduce postoperative analgesics consumption [54] and was beneficial on postoperative pain after thoracotomy, sternotomy, total knee arthroplasty, pulmonary surgery, and post-cesarean pain [55].

Acupuncture is another potential strategy to reduce pain in the postoperative period and was effective against controls in a randomized control trial on inguinal hernia repair [56]. Recent literature has been focused also on the use of electrical acupuncture, which was demonstrated to be more effective than traditional acupuncture in a randomized control trial on 121 patients requiring thyroid surgery [57].

Among other non-pharmacological interventions, sitz baths and moxibustion have been used after hemorrhoidectomy, [58] while aromatherapy was successfully used in pain relief after cesarean section and laparoscopic adjustable gastric banding, demonstrating that pain after surgery following inhalation of lavender essence was significantly decreased compared with the placebo groups [58, 59]. A 2016 systematic review and meta-analysis found that aromatherapy was effective in reducing pain scores in patients with acute and chronic pain, though the authors specify that the relatively few studies with substantially different protocols analyzing different types of pain may limit the strength of their conclusions [60].

Another potentially underestimated non-pharmacological strategy may involve the use of focussed preoperative patient education programs. A recent systematic review in orthopedic surgery concludes that multimodal preoperative education programs hold promise as an effective strategy to limit opioid consumption in the postoperative period [61]. In gynecological and abdominal surgery patients, preoperative counseling on postoperative pain management and on opioid medication adverse effects has been shown to reduce the consumption of opioids, with equivalent pain scores and medication refill requests [62].

Currently, a growing body of scientific evidence suggests that non-pharmacological strategies may have substantial effects in the treatment of both acute and chronic pain, though more large and well-designed studies are needed to reach a consensus regarding opioid-sparing analgesia in cancer surgery. Furthermore, among non-pharmacological strategies, other potential treatment modalities, such as relaxation, imagery/hypnosis, cognitive behavioral therapy/coping skills training, and meditation have not been studied extensively in the post-surgical setting and may also warrant future investigation [63].

### **“**Opioid-free anesthesia” and unintended consequences

As a reflexive response to the opioid epidemic, there have been a number of “opioid-free anesthesia” protocols shared within the anesthesia community [64]. In general, these approaches specifically target the avoidance of one class of medications and focus only on the time that patients spend within the operating room walls [65].

As anesthesiologists and acute pain medicine specialists, it is critical to acknowledge the important role of opioids in perioperative pain management, especially in the context of treating visceral pain [66]. While avoiding the potentially harmful effect of opioids is important, opioids may be prescribed safely by anesthesiologists and surgeons in the perioperative period when following appropriate guidelines [67]. Seven principles of acute perioperative pain management have been endorsed by 14 medical organizations in the USA and were recently published [68]. These principles emphasize the routine use of multimodal analgesia with the primary goal of improving the quality of pain management. Multimodal analgesic regimens frequently include non-pharmacologic interventions, non-opioid systemic analgesics, and local anesthesia and regional block techniques [68]. Patients’ requirement for opioids reliably decreases with the effective use of multimodal analgesia, but this is a side benefit while the goal is better perioperative pain management.

The deliberate restriction of opioids when they may be indicated, limiting them to the time when patients are in the operating room, may have unintended consequences. In a study of patients who underwent major non-cardiac surgery, patients assigned to an intraoperative opioid-free anesthesia protocol featuring dexmedetomidine experienced more postoperative side effects such as bradycardia, hypoxemia, and prolonged recovery than the opioid-exposed group [23]. Numerous experts, including the authors of the Centers for Disease Control and Prevention Chronic Opioid Prescribing Guideline [69], have expressed concern about the inappropriate restriction of opioids for acute pain [70–72]. There is no evidence that opioid-free anesthesia protocols decrease long-term postoperative opioid use.

An appropriate opioid stewardship plan must extend beyond the immediate intraoperative period [65, 67, 72]. It does not make sense to avoid opioids when patients are under anesthesia, only to then expose them to opioids as soon as they arrive in the postanesthesia care unit and during the rest of their hospital stay, and finally discharging them home with an arbitrary number of opioid tablets. Opioid-sparing multimodal analgesia must be continued after surgery for all patients on an inpatient and outpatient basis [73]. To date, only programs that have incorporated post-discharge opioid stewardship with individualized prescribing and tapering have demonstrated decreases in outpatient opioid use [74, 75].

### Future insights

Over the past two decades, opioids have increasingly been prescribed for the treatment of various chronic pain conditions and during the perioperative period. Opioids have multiple side effects, especially in elderly patients. Recent studies have also shown that the use of opioids in patients undergoing surgery is responsible for worse outcomes, an increase in the length of hospital stay, and an increase in healthcare costs [76].

OFA has gained in popularity as a way to enhance early recovery and to spare opioids for the postoperative period. While opioid-sparing strategies are recommended, little data exists on the feasibility of OFA in routine practice. However, promising results were obtained with OFA strategies, as described above. In the era of ERAS protocols, the use of multimodal analgesia with combinations of analgesic drugs and local regional anesthesia techniques is imperative [77, 78]. All this is in the effort to minimize the consumption of opioids and their side effects, moving from a present of opioid sparing to a future of opioid-free anesthesia [79, 80].

## Conclusions

Choosing the best anesthesia protocol for a patient undergoing surgery for cancer is not an easy task and the literature provides no definitive answers. However, in cancer patients, a pragmatic approach can be suggested considering the available options and the feasibility in the different settings. When possible, OFA strategies should be considered while ensuring that optimal pain management is achieved. Opioid administration may be needed but should be used in the lowest effective doses as part of multimodal analgesia. Considering the benefits and low risks, comprehensive patient education programs should be considered, as well as other non-pharmacological interventions when applicable. More well-designed studies are needed to reach a definitive conclusion on OFA in cancer surgery.

## Data Availability

Data sharing is not applicable to this article as no datasets were generated or analyzed during the current study.

## Abbreviations

ERAS: Enhanced Recovery After Surgery
NMDA: N-methyl-d-aspartate
OFA: Opioid-free anesthesia
PONV: Postoperative nausea and vomiting
TENS: Transcutaneous electrical nerve stimulation
